# Perioperative strategy and outcome in giant retroperitoneal dedifferentiated liposarcoma—results of a retrospective cohort study

**DOI:** 10.1186/s12957-020-02069-2

**Published:** 2020-11-12

**Authors:** Robert Bachmann, Franziska Eckert, Daniel Gelfert, Jens Strohäker, Christian Beltzer, Ruth Ladurner

**Affiliations:** 1 Department of General, Visceral and Transplantation Surgery, University of Tübingen, Hoppe-Seyler Str. 3, 72076 Tübingen, Germany; 2 Department of Radiation Oncology, University of Tübingen, Hoppe-Seyler Str. 3, 72076 Tübingen, Germany

**Keywords:** Radiation therapy, Recurrence analysis, Retroperitoneal soft tissue sarcoma, Surgery

## Abstract

**Background and objectives:**

Retroperitoneal liposarcoma (RPLS) are common soft tissue sarcomas of adulthood. The aim of this study is to show resectability of even giant liposarcomas and to identify factors associated with recurrence and survival in primary retroperitoneal liposarcomas.

**Methods:**

We retrospectively reviewed the records of patients with retroperitoneal liposarcoma. Seventy-seven patients met inclusion criteria. Out of these 10 patients with primary giant, dedifferentiated retroperitoneal liposarcomas were operated with en bloc compartment resection with intention of radical resection. Treatment consisted of neoadjuvant radiochemotherapy and surgical resection or surgical resection.

**Results:**

In 6 patients, neoadjuvant radiochemotherapy was performed; 3 patients were treated with surgical resection alone and 1 patient received adjuvant chemotherapy. The median diameter of tumor size was 360 mm (300 to 440 mm). Operative outcome showed complete resection in all 10 patients. Local tumor free survival was in median 19 month. Tumor recurrence was seen in 3 of 4 patients (75%) without neoadjuvant radiochemotherapy, and in 2 of 6 patients (33%) after neoadjuvant radiochemotherapy in 2 years follow-up.

**Conclusion:**

Even in case of giant retroperitoneal liposarcoma, complete resection is possible and remains the principal treatment. The rate of recurrence was improved in patients with neoadjuvant radiochemotherapy.

## Synopsis

The treatment of dedifferentiated retroperitoneal sarcomas of remarkable size is a challenge. On the one hand, optimal therapeutic treatment requires the combination of neoadjuvant radiochemotherapy and surgical resection with oncological radicality. On the other hand, combined treatment means an enormous somatic burden.

## Introduction

Liposarcomas are neoplasms of mesodermic origin derived from adipose tissue. It is the most common tumor entity of the retroperitoneum but represents less than 1% of all malignant tumors [1]. Retroperitoneal soft tissue sarcomas account for about 15% of all soft tissue sarcomas [2]. The two most common retroperitoneal types are well-differentiated (WDLPS) and high-grade dedifferentiated (DDLPS) liposarcomas [3]. The average diameter of the tumor is 20–25 cm with a weight of 15 to 20 kg [4].

Surgical resection is considered the most important treatment of retroperitoneal liposarcoma. Late presentation, anatomical conditions, and invasion of adjacent structures aggravate the therapy. Therefore, in most of the patients, optimal treatment requires a more aggressive surgical approach, including multiorgan resection. Multimodality treatment is increasingly used in most sarcoma centers. The treatment in combination with radiotherapy trends towards use of preoperative radiotherapy. Adjuvant treatments have limited efficacy [5].

According to the eighth edition of the AJCC Cancer Staging Manual, the staging of sarcomas of the retroperitoneum is performed using following criteria: tumor size, nodal status, histologic grade, metastasis, and anatomic primary tumor site. All tumors above 15 cm are tumors of the T4 category. The definition of giant in our study was therefore chosen for tumors beyond twice of pT4 category. These “giants” are normally filling out the complete, expanded retroperitoneum with displacement of the intraabdominal organs to the contralateral site.

The objective of this study is to show the actual treatment and experience in the treatment of patients with giant dedifferentiated retroperitoneal liposarcoma in combination with neoadjuvant radiochemotherapy. To the best of our knowledge, this evaluation is the first document regarding giant retroperitoneal liposarcoma and its clinicopathological characteristics, evaluation of neoadjuvant radiochemotherapy, and survival.

## Methods

### Patients

Medical records of all consecutive patients who underwent surgery at the University hospital Tübingen for retroperitoneal liposarcoma with curative intent from January 2003 to December 2018 were identified and retrospectively analyzed from a prospectively maintained database. All patients with retroperitoneal liposarcoma and a tumor diameter above 30 cm were included. Exclusion criteria were recurrent and metastatic tumors. Overall survival (OS) and disease-free survival were reviewed.

Patient data were compared between patients with or without recurrence within 48 months after surgery. Risk factors were determined using logistic regression analysis. When a patient underwent several operations for retroperitoneal LPS, the first operation performed at our center was included for analysis.

### Data collection

Data on patient demographics and treatment parameters were reviewed. Recurrence data related to the first operation performed at our center were assessed. Surgical factor data such as resected organs and completeness of resection were collected based on operation records. Size of tumors, histologic differentiation, margin status, and the presence of organ invasion were assessed. Data on radiotherapy (RT) were also collected.

### Data analysis

The time point for recurrence was set as the first time when imaging findings were suspicious for recurrence of dedifferentiated liposarcoma. Even when findings were suspicious but not definite for recurrence, the first time the finding was observed was set as the time of recurrence if the finding was confirmed as a recurrent tumor on the concurrent CT scan. Radiotherapy was divided into no radiotherapy, adjuvant and neoadjuvant radiotherapy. Kaplan-Meier survival was used to estimate 1- and 2-year disease-free survival.

## Results

### Study cohort

In the defined time period, 77 patients were identified with primary retroperitoneal liposarcoma. Histology, grading, and tumor diameter were analyzed from the medical records. Ten consecutive patients with giant dedifferentiated liposarcoma were identified and were included in the retrospective evaluation.

### Patient characteristics

The median age was 57 years (range 40 to 76). Median tumor size was 360 mm with a range from 300 to 440 mm. Treatment consisted of surgical resection in 4 patients and multimodal treatment with neoadjuvant radio-(chemo)therapy and surgical resection in 6 patients.

### Neoadjuvant radiotherapy

Six of the reported patients were treated with neoadjuvant radiotherapy or neoadjuvant multimodal treatment. Five patients received ifosfamide chemotherapy concomitant to radiotherapy; one patient was treated with a trimodal approach encompassing radiotherapy, chemotherapy, and locoregional hyperthermia.

Radiotherapy was planned to 45 Gy in 1.8 Gy fractions for five of the six patients. All but the first patient were treated with intensity-modulated radiotherapy (IMRT). Contoured organs at risk (OARs) included kidney, liver, spinal cord, small and large bowel, and bladder. Gross tumor volume (GTV) was contoured taking into account pretreatment MR imaging and clinically available information. Planning target volume (PTV) margins were calculated with a > 5 mm expansion around the GTV with or without creating a clinical target volume (CTV) taking into account anatomical borders. Mean GTV size was 7715 cm^3^ (range 1386–13,916 cm^3^), mean PTV size was 10,796 cm^3^ (range 4030–18,707 cm^3^).

**Fig. 1:**
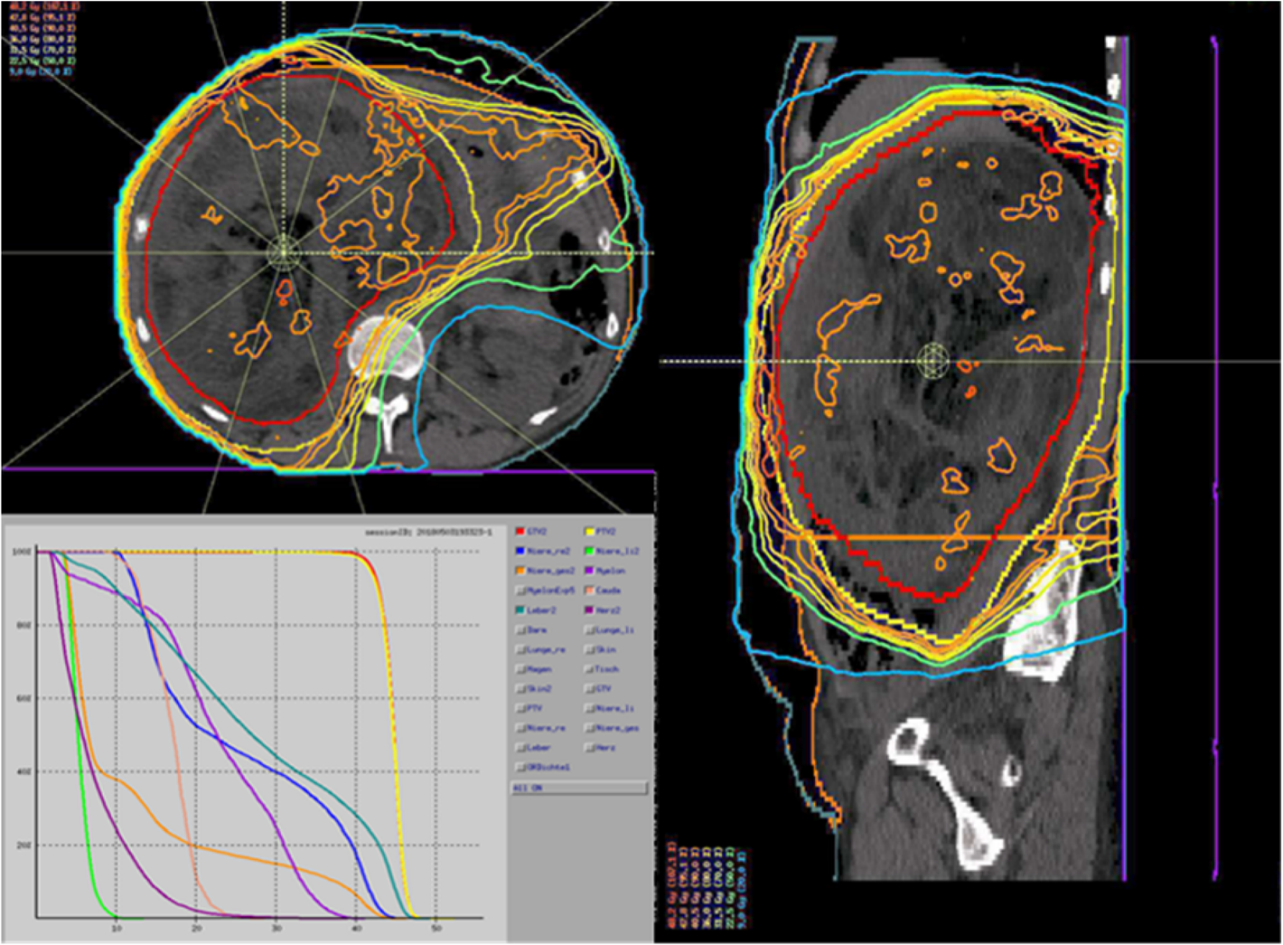
Representative sections (axial and sagittal) of a radiotherapy plan show the GTV (red) and PTV (yellow) as well as isodose lines. Despite inhomogeneity in the dose distribution planning, objectives were met. The axial sections show maximal sparing of the left kidney, which is outside the 9 Gy isodose (blue). The dose-volume histogram (DVH) indicates a good dose coverage for both the GTV (red) and PTV (yellow) and respecting of all dose constraints for organs at risk with < 45 Gy maximum dose in 2% of the volume for the spinal cord (dark purple) and low dose to the left kidney (light green)

The planning objective was a coverage of the GTV with a minimal dose (for conventional radiotherapy planning) or dose to 98% of the contoured volume (D98) for IMRT of > 90% of the prescribed dose. Kidney sparing radiation was aimed at for both kidneys or contralateral kidney if the planned surgical procedure included unilateral nephrectomy. Maximal dose or D2 to the small bowel and the spinal cord was limited to 45 Gy. Treatment planning was possible for all patients for whom neoadjuvant radiotherapy had been suggested by the interdisciplinary tumor board without significant risk of severe long-term side effects (Fig. 1).

### Surgical and oncological outcome

Tumor resection was complete in 9 patients. One patient pretreated with radiochemotherapy showed abdominal metastases during primary operation. The overall 1 and 2 year survival rate was 100 and 90%, respectively. Tumor recurrence was seen in 5 patients. The patient with abdominal metastases suffered from early tumor progress. This patient was therefore excluded from further evaluation. From the remaining five patients with neoadjuvant radiochemotherapy 1 patient suffered from tumor recurrence (20%) within the 2-year follow-up period. Out of the four patients with surgical resection alone, three had tumor recurrence (75%) within the 2 years follow-up (median 16 month). Median time to tumor recurrence was 18 month (Table 1).

**Table 1:** The perioperative treatment and postoperative outcome parameters

Parameter	Variable	Value (***n***)
Gender	Female	5
	Male	5
Age	Years, median (range)	58 (40–76)
Radiochemotherapy	Neoadjuvant (with additional hyperthermia)	6 (1)
	Adjuvant	0
	None	4
Chemotherapy	Adjuvant	1
	None	3
Origin	Retroperitoneal	
	Left	4
	Right	6
Operative procedures performed	Laparotomy, midline	10
	En bloc compartmental resection + 0 organs	2
	En bloc compartmental resections + 1 organ	3
	En bloc compartmental resections + 2 organs	4
	En bloc compartmental resections + 3 organs	1
Resected organs	Kidney	4
	Intestine, large	6
	Adrenal gland	2
	Orchiectomy	2
Operation time	Minutes, median (range)	253 (165–458)
Specific perioperative complications:	Major	2
	Necessitating laparotomy	2
	Anastomotic insufficiency bowel	1
	Minor	2
	Urinary tract infection (Candida)	1
	Diarrhea (*C. difficile*)	1
Hospital stay	Duration (days)	15.2 (11–21)
Pathologic dimension	Median	360 (300–440)
	> 300 and < 350 mm	4
	> 350 and < 400 mm	4
	> 400 mm	2
Surgical margins	Wide (R0)	3
	Marginal (R1)	7
	Intralesional (R2)	0
Histology	Dedifferentiated	10 (100%)
Tumor response	Radiologic (RECIST)	
	Regressive	0
	Stable	6 (100%)
	Progressive	0
	Histologic (% tumor vitality)	
	< 20	0
	20–30	1
	30–60	3
	60–90	2
	> 90	0
Tumor relapse	Site of recurrence	
	Local	3
	Local metastatic (abdominal/peritoneal)	2
	Metastatic (pulmonal/cerebral)	1

## Discussion

This is the first study to report the treatment outcome of a cohort of giant dedifferentiated retroperitoneal liposarcoma. As this type of cancer is very rare, information about treatment and outcome is very limited. Prospective studies are not available. Treatment of these patients is current guided by surgical assessment and expertise based on the principals of treatment of normal retroperitoneal liposarcomas. Up to date, there are just several case reports of giant retroperitoneal liposarcoma available [6, 7]. Only few surgeons and radiooncologists gain enough treatment experience of these patients; therefore, some of the patients receive suboptimal treatment with possible unsatisfactory inferior outcomes.

Surgery is the mainstay of treatment for non-metastatic retroperitoneal liposarcoma. Macroscopic complete resection should be aimed for, often requiring en bloc removal of adjacent structures such as the abdominal wall, psoas, or paravertebral muscles.

The treatment of the presented study was in the line of the current guidelines [8, 9].

Complete surgical resection is the cornerstone of therapy, and it is the most consistent prognostic factor [10]. Size alone does not contraindicate complete resection of giant retroperitoneal liposarcoma, which is currently the only potentially curative treatment. In specialized centers, most of the liposarcomas can be surgically removed, which is consistent with the consensus management of RPLS in the adult [11]. In dedifferentiated retroperitoneal liposarcoma, resection alone might be undertreatment because of the aggressiveness of the disease and the combination with neoadjuvant radiochemotherapy is necessary for local control. The survival impact of neoadjuvant radiochemotherapy specifically for patients with giant retroperitoneal liposarcoma is not defined. However, the only randomized trial (STRASS EORTC 62092), which compared preoperative radiotherapy plus surgery to curative-intent surgery alone in the treatment of non-metastatic retroperitoneal sarcoma demonstrated beneficial effects of preoperative radiation therapy concerning local relapse in the subgroup of the retroperitoneal liposarcoma [12]. The concept of neoadjuvant radiochemotherapy allows the radiotherapy oncologist to better define the target volume and avoid unnecessary irradiation of adjacent normal tissues. This limits the prescribed radiation dose and decreases the amount of radiotherapy that normal structures receive in comparison to postoperatively administered radiotherapy [13, 14]. Additionally, preoperative radiotherapy might improve rates of surgical margin-negative resection, either by facilitating complete resection or by sterilizing the margin at risk [15]. To obtain clear surgical margins and considering the highly aggressive nature of retroperitoneal liposarcomas and their propensity for local recurrence and distant metastasis, complementary adjuvant/neoadjuvant therapy may be required [16]. However, adjuvant therapy including either chemotherapy and/or radiotherapy is of little study-proven value and just benefit a minority of patients [17, 18]. The role of chemotherapy is not as well defined as is the role of radiation therapy. Several trials were unable to determine conclusively whether doxorubicin-based adjuvant chemotherapy benefits adults with resectable soft tissue sarcomas [19]. The retroperitoneal sarcoma subtypes that might benefit the most from chemotherapy are high-grade (G3 according to the Fédération Nationale des Centres de Lutte Contre Le Cancer grading system), dedifferentiated liposarcoma, leiomyosarcoma, and undifferentiated pleomorphic sarcoma, which tend to be chemosensitive [20]. Doxorubicin remains the mainstay of systemic therapy in the management of locally advanced and metastatic soft tissue sarcoma. Another treatment modality with growing attention is neoadjuvant chemotherapy as a radiosensitizer in conjunction with radiation therapy. A recently published phase I/II study assessed the long-term outcomes of retroperitoneal liposarcoma patients treated with three cycles of high-dose, long-infusion ifosfamide in conjunction with radiotherapy prior to surgery [21]. Following the STRASS, another randomized study is in progress. STRASS 2 is going to address the role of neoadjuvant chemotherapy (Surgery With Our Without Neoadjuvant Chemotherapy in High Risk RetroPeritoneal Sarcoma (STRASS2)). In a phase II study, the effect of trabectedin in advanced retroperitoneal leiomyosarcoma and well-differentiated/dedifferentiated liposarcoma is under evaluation (EudraCT number 2012-005428-14). However, systemic chemotherapy has until now a limited role in the treatment of RPLS. The combination of radiotherapy with other compounds, such as trabectedin or molecular target agents, is currently under investigation. The benefit of additional intraoperative radiotherapy aiming increased radiation doses in regions of close or positive resection margins remains questionable [22].

Although many sarcoma centers routinely use radiotherapy as an adjunct in the management of patients with retroperitoneal liposarcoma, there is no consensus with regard to indications or benefit. The role of radiotherapy in the treatment of retroperitoneal sarcoma remains under debate. Increasing numbers of studies are showing the potency of neoadjuvant radiotherapy in dedifferentiated retroperitoneal liposarcoma. The feasibility and tolerance of preoperative radiotherapy was shown without increase morbidity or mortality following resections [23]. However, the presented series are dealing with smaller tumor diameters. In this regard, there are recent studies of retroperitoneal liposarcoma with radiation therapy and a median tumor size between 12.8 cm [24], 14,5 cm [25], 13,6 cm [26], 15 cm [27], and 15,4 cm [28].

The main issue in the treatment of the differently differentiated retroperitoneal liposarcomas is the separation of the two main risks of tumor relapse—local recurrence and distant metastases [29]. In this study, large tumor size, high grade, and incomplete tumor resection were predictors of local recurrence. The role of radiotherapy may be in grade 1 to 2 dedifferentiated liposarcomas (DDLPD) as they tend to have a higher rate of local recurrence rather than a risk of distant metastases. The biggest study to report on this special focus was presented by Haas et al. Their patient collective with grade 1 and 2 dedifferentiated retroperitoneal liposarcomas had a median tumor size of 27 cm (19.0–35.0, first and third quartile). In the dedifferentiated subgroup, radiotherapy was predominantly administered to patients with smaller tumors. Only 31.1% and 16.1% of the patients with a tumor size of 30 to 40 cm or > 40 were treated with radiotherapy [30].

The use of radiotherapy in patients with grade 1 and 2 dedifferentiated retroperitoneal liposarcoma may be beneficial regarding local control. In grade 3 dedifferentiated retroperitoneal liposarcoma, the risks of distant metastases may outweigh the benefits of radiotherapy. Our study focused on supplement radiotherapy even for the giant dedifferentiated liposarcomas. Therefore, the individualized indication of radiotherapy has a rationale in grade 1 and 2 dedifferentiated retroperitoneal liposarcoma and also in the giant as our study shows.

The results of our study are limited due to the retrospective study design and the small number of patients. Nevertheless, the strength of this study is to be the first evaluation of patients with the same tumor (de-)differentiation, the giant tumor volume in every patient, the clear advantage of radiotherapy and the same surgical concept. Furthermore, the results of this study are in the line with the treatment of normal-sized dedifferentiated retroperitoneal liposarcoma. If the results of larger studies are compared, the benefit of radiochemotherapy could only be suspected so far, since these subgroups were not further evaluated. Thus, very limited data exists on giant retroperitoneal liposarcomas with dedifferentiated histology. This is the first study to solely focus on perioperative outcome in patients with giant liposarcomas with or without neoadjuvant radiochemotherapy. While the small sample size is the main limitation of this study, we are currently forced to treat this patient subgroup based on only case reports. Our patient collective of ten patients with comparable histology shows that even the largest of these tumors can be successfully treated with neoadjuvant radiochemotherapy followed by complete resection thus offering a chance of long-term survival. Given the rarity of the disease, we are at the beginning of evaluating long-term outcomes of these patients. However, a 2-year survival rate is a strong indicator to initiate trials to substantiate our results. Therefore, we are in need for a prospective multicenter trial.

## Conclusions

Giant retroperitoneal liposarcomas can be removed by a multidisciplinary team in specialized centers after meticulous planning. Neoadjuvant radiotherapy improved the rate of recurrence and may improve the rate of survival in patients undergoing surgery for retroperitoneal liposarcoma, particularly those with high-risk pathological features.

## Data Availability

Data and material supporting our findings are presented on request.
